# Water, sanitation, and hygiene access among people who inject drugs in Tijuana and San Diego in 2020–2021: a cross-sectional study

**DOI:** 10.1186/s12939-024-02163-x

**Published:** 2024-04-22

**Authors:** Alhelí Calderón-Villarreal, Lourdes Johanna Avelar Portillo, Daniela Abramovitz, Shira Goldenberg, Shawn Flanigan, Penelope J. E. Quintana, Alicia Harvey-Vera, Carlos F. Vera, Gudelia Rangel, Steffanie A. Strathdee, Georgia L. Kayser

**Affiliations:** 1 Department of Family and Preventive Medicine, University of California San Diego (UCSD), San Diego, California, USA; 2 School of Public Health, San Diego State University (SDSU), San Diego, California, USA; 3 Benioff Homelessness and Housing Initiative, School of Medicine, University of California, San Francisco, California, USA; 4 Division of Global Health, Herbert Wertheim School of Public Health and Human Longevity Science, UCSD, San Diego, California, USA; 5 Department of Medicine, Division of Infectious Diseases and Global Public Health, UCSD, San Diego, California, USA; 6 School of Public Affairs, SDSU, San Diego, California, USA; 7 Universidad de Xochicalco, Tijuana, Baja California, Mexico; 8 El Colegio de la Frontera Norte, Tijuana, Baja California, Mexico; 9 Border Health Commission, Tijuana, Baja California, Mexico

**Keywords:** WASH, WASH insecurity, PWID, US-Mexico border, Homelessness, Health inequalities

## Abstract

**Background:**

Water, sanitation, and hygiene (WASH) access is critical to public health and human dignity. People who inject drugs (PWID) experience stigma and structural violence that may limit WASH access. Few studies have assessed WASH access, insecurity, and inequities among PWID. We describe WASH access, social and geographic inequalities, and factors associated with WASH insecurity among PWID in the Tijuana-San Diego metropolitan area.

**Methods:**

In this cross-sectional binational study, we interviewed PWID (age 18+) in 2020–2021 about WASH access and insecurity. City of residence (Tijuana/San Diego) and housing status were considered as independent variables to describe key WASH access outcomes and to assess as factors associated with WASH insecurity outcomes. Measures of association between outcomes and independent variables were assessed using log modified-Poisson regression models adjusting for covariates.

**Results:**

Of 586 PWID (202 Tijuana; 384 San Diego), 89% reported basic access to drinking water, 38% had basic hand hygiene, 28% basic sanitation, and 46% access to bathing, and 38% reported recent open defecation. Participants residing in Tijuana reported significantly higher insecurity in accessing basic drinking water (aRR: 1.68, 95%CI: 1.02–2.76), basic hygiene (aRR: 1.45, 95%CI: 1.28–1.64), and bathing (aRR: 1.21, 95%CI: 1.06–1.39) than those living in San Diego. Participants experiencing unsheltered homelessness experienced significantly higher insecurity in accessing basic drinking water (aRR: 2.03, 95%CI: 1.07–3.86), basic sanitation (aRR: 1.68, 95%CI: 1.48, 1.92), bathing (aRR: 1.84, 95%CI: 1.52–2.22), and improved water sources for cleaning wounds (aRR: 3.12, 95%CI: 1.55–6.29) and for preparing drugs (aRR: 2.58, 95%CI: 1.36–4.89) than participants living in permanent housing.

**Conclusion:**

WASH access among PWID in the Tijuana-San Diego metropolitan area was low by international standards and lower than the national averages in both countries. Homelessness was significantly associated with WASH insecurity in this population. Concentrated efforts are needed to guarantee continuously available WASH services for PWID—especially those who are unsheltered.

**Supplementary Information:**

The online version contains supplementary material available at 10.1186/s12939-024-02163-x.

## Background

In recent decades, the Millennium Development Goal for Water and Sanitation (MDG 7), the Sustainable Development Goal for Water and Sanitation (SDG 6), and the Human Right to Water and Sanitation have helped to improve awareness and increase water, sanitation and hygiene (WASH) access at the household level worldwide [1]. Yet, vulnerable and marginalized populations continue to lack access to these basic services [2]. Basic WASH access helps prevent water-related diseases such as malnutrition, myocarditis, viral, protozoan, helminth or bacterial infections, and even multidrug-resistant organisms [3], which can cause death or compromise people’s health. Furthermore, WASH access and insecurity became critically important during the recent pandemic outbreaks. Mpox virus control includes good hygiene practice and SARS-CoV-2 may be shed in feces [4, 5].

We utilized basic WASH service definitions based on the World Health Organization (WHO) & United Nations International Children’s Emergency Fund (UNICEF) Joint Monitoring Program (JMP) definitions [6]. Therefore, in this study, we defined WASH insecurity as the lack of at least basic access to drinking water, sanitation, hygiene, and improved water for bathing, drug preparation and cleaning of wounds. WASH insecurity experiences can increase exposure to water-related health risks [7–9]. People living in areas with WASH insecurity could be more likely to acquire and transmit water-related diseases, especially those experiencing intersectional vulnerabilities, such as people experiencing homelessness and engaging in substance use [4, 9–11].

People who inject drugs (PWID) are a marginalized population; lack of access to safe water for the preparation of injections and for cleaning wounds and abscesses increases their vulnerability [3, 12, 13]. Drug preparation with contaminated water sources can lead to fungal and bacterial infections, especially if the water is not boiled or treated [12, 13]. Contaminated water sources and inconsistent hand hygiene are associated with injection-related injury and diseases, such as skin and soft tissue infections (SSTI), especially abscesses, among PWID [12, 14, 15]. Abscesses and vascular damage are common injuries among PWID, which can be life-threatening, leading to necrotizing fasciitis, gangrene, septic shock, endocarditis, and death [14]. However, little research has focused on the use of water for cleaning wounds and abscesses, and for preparing drugs for injection among PWID [12, 14, 16].

Similarly, few studies have addressed the personal hygiene needs of PWID, especially basic hand hygiene practices that are important in preventing enteric and respiratory infectious diseases, such as SAR-CoV-2 [17]. During the COVID-19 pandemic, an ethnographic study found that stigmatization and criminalization increased PWID’s vulnerability to homelessness and lack of basic services [18] – such as adequate WASH access [17] – resulting in higher risk of water-related infectious diseases among PWID.

Based on research gaps on WASH access among PWID – and particularly by housing status, our study explores WASH access among PWID in the Tijuana–San Diego metropolitan area. Collectively Tijuana and San Diego are home to a large population of PWID (~ 10,000 in Tijuana and ~ 21,800 in San Diego), many of whom are experiencing homelessness or have been deported from the US [16, 19–21]. PWID living in this United States (US)-Mexico border region are particularly susceptible to infectious disease and chronic health problems, including SSTI [47% prevalence among PWID and who were experiencing unsheltered homelessness in Tijuana] [22, 23], and septic shock [18% of deaths among PWID in Tijuana from 2011 to 2018] [21]. Intersectional vulnerabilities such as experiences of homelessness and drug use are also risk factors for methicillin-resistant *Staphylococcus aureus* infection, a leading cause of hospitalization among these populations [24], and can be associated with WASH insecurity. An intersectionality approach based on “clusters of disadvantages” is used to analyze intersection of gender and race systems of oppression [25]. Yet, intersectionality can be extended to any oppression system, such as substance use and homelessness or geographic inequalities, as we briefly incorporated in this study.

To characterize the existing gaps in WASH among this marginalized population and to identify inequities in social and environmental determinants of health, our study described WASH access outcomes, while also examining social and geographic inequalities, and factors associated with WASH insecurity among PWID in the Tijuana-San Diego metropolitan area in 2020–2021.

## Methods

### Study design and dataset

This was a cross-sectional study based on data from interviewer-administered surveys [conducted computer-assisted programmable interviews – CAPI] in the participants’ language of preference [English or Spanish] to PWID in the Tijuana–San Diego metropolitan area as part of the *La Frontera* binational cohort study. Data were collected by trained interviewers in 2020–2021 using street outreach and mobile vans in Tijuana and San Diego, as previously described in Strathdee et al.,2021 [26]. Eligibility criteria included individuals aged ≥18 or older, who reported injecting drugs within the last month in San Diego, Tijuana, or in both cities. Only participants who completed the WASH component questionnaire collected 1 week after the baseline survey formed part of this study. Participants’ informed consent was obtained, and monetary reimbursements were provided. This study received ethics approval from the institutional review boards at UCSD and UCLA in the United States (IRB# 800668), and Xochicalco University in Mexico (IRB # 191390).

### Independent variables and covariates– social and geographic variables

Social and geographic independent variables of interest included city of residence and housing status. Housing status was classified as permanent housing, sheltered or unsheltered homelessness [27, 28], based on people’s reporting of the places where they have lived or slept, and the main place where they slept in the past 6 months. Permanent housing included people who reported sleeping in their parent’s, their own, their spouse’s/sex partner’s, family or friend’s house or apartment. Sheltered homelessness included people who reported sleeping in migrant worker’s camp, asylum seekers shelter, shelter/welfare residence, workplace, rented room (hotel, motel, or other rooming house), deportee shelter/camp, correctional institution (jail, prison, detention center), drug treatment center, medical care facility (i.e., hospital, hospice, or nursing home), or rented garage. Unsheltered homelessness included people who reported sleeping in their car, bus, truck, or other vehicle, abandoned building, on the streets, beach, parks, canal, woods, and shooting gallery.

Other covariates described were age, weight, income, gender identity (including man, woman, and transgender), and engagement in sex work. Sex work was defined as a source of income described as ‘prostitution or sex work’ in the past 6 month.

### Outcomes - WASH variables

WASH access assessment included questions related to drinking water, sanitation, and hygiene (including handwashing, cleaning of wounds/abscesses, and showering/bathing) access in the past 6 months. First, drinking water access was assessed by identifying the main water source used, availability of drinking water sources, time spent collecting water, and the number of glasses of water and other beverages consumed in the last week. Second, sanitation access was determined by identifying the main sanitation facility used (i.e., open defecation, type of toilet/latrine), availability of the facility, whether the facility was shared with other households, the privacy (i.e., toilet with door that locks), the functionality, and experience of violence/harassment using a toilet. Hygiene access was measured with questions on availability of soap and water, water sources used for handwashing, availability of alcohol-based rub, and handwashing practices (after a bowel movement, before eating, and before preparing food). Additionally, we described water sources used for cleaning wounds and abscesses, for preparing drugs for injection, and showering/bathing practices in the last week (e.g., frequency and water source type). We included changes in water and soap availability since the COVID-19 pandemic (March 2020) and a measurement of cost (in USD) spent to access WASH services in the last month.

**Fig. 1:**
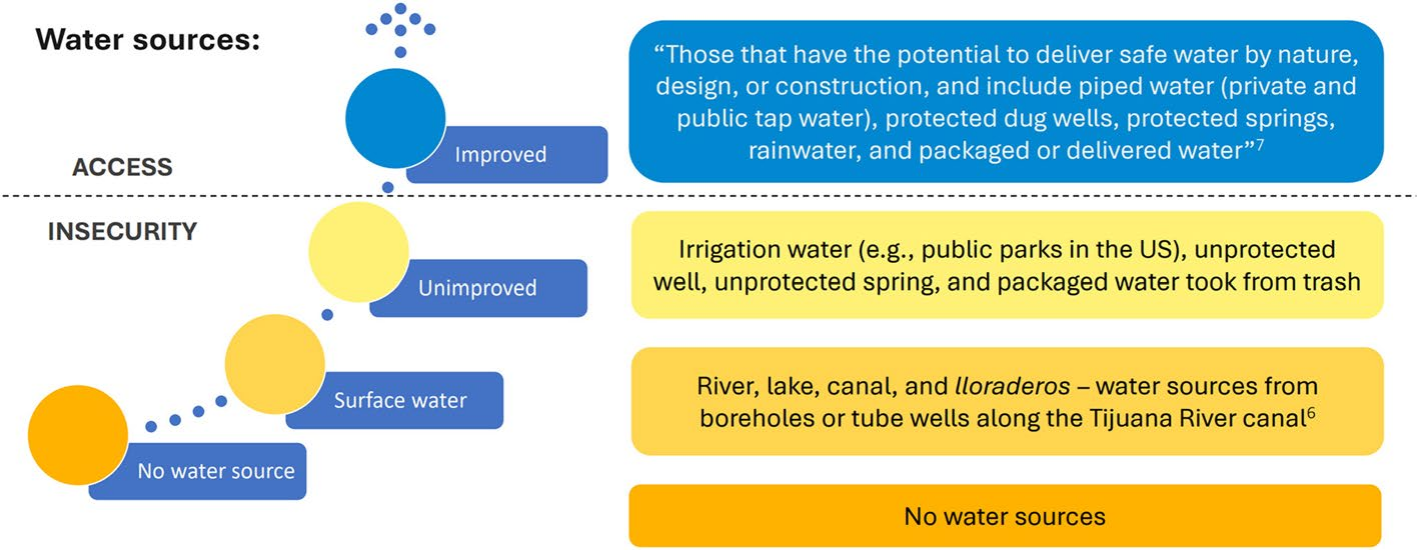
Water sources classification based on WHO/UNICEF Joint Monitoring Program

Basic WASH access variables were operationalized according to the WHO/UNICEF JMP definitions [6]. Basic drinking water access included the use of an improved water source in ≤30 minutes away roundtrip. Water sources definitions are described in Fig. 1. Basic sanitation access was defined as the use of improved facilities not shared with other households. Improved sanitation facilities were those which separate excreta from human contact, including flush/pour flush toilets connected to piped sewer systems, septic tanks, or pit latrines; pit latrines with slabs, and composting toilets. Basic hygiene access was defined based on the availability of a handwashing facility with soap and water. JMP definitions for water sources were used also for handwashing, bathing, cleaning wounds and abscesses, and preparing drugs for injections [6]. Bathing was defined as having > 4 bathes per week with improved water sources. Additionally, a set of complementary basic WASH variables were included to measure continuity of basic WASH services (i.e., always/24 hours service availability), and for basic hand hygiene we also measured the use of improved water sources and soap availability.

Key WASH outcomes were selected to describe social and geographic inequalities in WASH access and to assess WASH insecurity factors associated with them. These included 1) basic drinking water, 2) basic sanitation, 3) basic hygiene, 4) bathing, and improved water source for 5) cleaning wounds/abscesses and 6) preparing drugs for injection. WASH insecurity outcomes were measured as the lack of these six key WASH access indicators.

### Data analyses

Of 612 participants in *La Frontera*, 586 (95.8%) completed the WASH survey and were included in analysis (Supplementary Table 1). All 26 participants who did not complete the WASH survey were from San Diego, and 25 of them were people experiencing homelessness. City of residence (*p* < 0.001) and housing status (*p* < 0.001) were significantly different between those who did and did not complete the WASH survey; there were no significant differences in survey completion based on gender (*p* = 0.555) and sex work status (*p* = 0.225).

Descriptive statistics were generated by estimating means and standard deviations (SD) along with medians and interquartile ranges (IQR) – according to their distribution – for the interval variables and frequencies and percentages for the categorical or binary variables. Number of glasses of water per day were described as a frequency and were used to estimate the daily water intake in milliliters per kilogram (ml/kg) of participant’s weight. Forty ml/kg were described as the recommended daily water intake [29]. Open defecation was described as both a binary variable and as a frequency (e.g., times a person openly defecated in the last week).

Inequality analysis included two components, the description of key WASH access outcomes by housing status and city of residence, and the assessment of those stratifiers as factors associated with key WASH insecurity outcomes. Inequalities accessing WASH were described as percentages of PWID who reported access to key WASH outcomes by city of residence and housing status. Inequalities assessment analyzed those social and geographic stratifiers as factors associated with outcomes of WASH insecurity (i.e., lack of WASH access). Measures of association between independent variables (stratifiers) and WASH insecurity outcomes were assessed using bivariate and multivariable analyses. We estimated crude (RR) and adjusted risk ratios (aRR) using a generalized linear model approach, leveraging a modified-Poisson distribution, and a log link, with a sandwich estimator to estimate robust standard errors [30]. Multivariable regression models included city of residence and housing status as independent variables and were adjusted for gender, and sex work as covariates. Two-way interactions were tested between independent variables to identify reference categories in each stratifier. Ninety-five percent confidence intervals (95% CI) were computed to identify significant results (alpha = 0.05). Data analyses were performed using R version 4.0.2.

## Results

Of the 586 participants who completed the WASH survey, 66% lived in San Diego and 34% in Tijuana in 2020–2021 (Table 1). The mean age of participants was 43 years old (±11), and mean weight was 70 kg (±14). Most were men (75%), and one person identified as transgender (0.2%). Sheltered and unsheltered homelessness was common among participants in both cities (78% in Tijuana; 56% in San Diego). Most of them reported that their main housing type did not change in the last 6 months (70%) and more than half (52%) experienced unsheltered homelessness at least once in the past 6 months. Nine percent of participants reported sex work in the last 6 months (30% of women and 5% of men). In Tijuana, 64% of the participants had <$175 USD/month income, and in San Diego, 49% had <$1000 USD/month income.

**Table 1:** Characteristics of 586 PWID participants in the Tijuana-San Diego metropolitan area in 2020–2021

Variables	Tijuana		San Diego		All	
	n	Value	n	Value	n	Value
Age^a^	202	44.5 (9.7)	384	42.6 (11.0)	586	43.2 (10.6)
Gender (%)						
Men	146	72.3	291	75.8	437	74.6
Women	55	27.2	93	24.2	148	25.3
Trans men	1	0.5	0	0	1	0.2
Housing status (%)						
Main housing in the last 6 months						
Permanent housing	44	21.8	168	43.8	212	36.2
Sheltered homelessness	66	32.7	59	15.4	125	21.3
Unsheltered homelessness	92	45.5	157	40.9	249	42.5
Unsheltered homelessness at least once in the last 6 months	102	50.5	201	52.3	304	51.9
Sex work in the las 6 months (%)						
No	162	80.2	370	96.4	532	90.8
Yes	40	19.8	13	3.4	53	9
Missing	0	0	1	0.3	1	0.2
Total	202	100	384	100	586	100

^a^ Mean (SD)

### WASH access

Eighty-nine percent of the participants had access to basic drinking water, and 56% had basic drinking water access ‘always available’ (24-hours a day) (Table 2). Participants spent a median of 4 minutes (range from 0 min to 12 hours) traveling to their main drinking water source and returning from it. More than half (51%) of the participants reported drinking a beverage other than water when they felt thirsty, such as soda (35%) and sweetened beverages (8%). It was common that participants felt thirsty without having access to drinking water every day/multiple times per day (20%) or multiple times per week (25%). Participants drank a median of four glasses (IQR: 2–6) of water per day, and 96.9% had a daily water intake under the medical recommendation, with a median 16.4 ml/kg (IQR: 7.7–23.3) water intake per day. Drinking water availability did not change after the onset of the COVID-19 pandemic (March 2020) for 77% of the participants; however, for 18% of individuals, access became more limited. Participants spent a median of $5 USD (IQR: $2.5, $10), ranging from $0.3 to $200 USD, on drinking water per month. For the previous 6 months, the main drinking water source was packaged water (48%) and tap water (44%) (Table 3). Six percent of the participants reported no access to water sources, and 5% used unimproved water sources or surface water for drinking at least once in the last 6 months.

**Table 2:** WASH access variables among 586 PWID in Tijuana-San Diego metropolitan area in 2020–2021

WASH indicator	n	Value	WASH indicator	n	Value
**Drinking water**			**Hygiene**		
Basic drinking water + availability (%)^c^	331	56.5	Basic hand hygiene + improved water source + soap availability (%)^c^	172	29.4
Basic drinking water (%)^a^*	524	89.4	Basic hand hygiene (%)^a^*	220	37.5
Improved water source for drinking (%)	542	92.5	Handwashing with water and soap availability (%)^c^	220	37.5
Drinking water collection time ≤30 min (%)	567	96.8	Improved water source for handwashing (%)	544	92.8
Time collecting drinking water (min)^b^	585	4 (1, 10)	Water source for handwashing availability (%)^c^	316	53.9
Drinking water availability (%)^c^	374	63.8	Soap availability (%)^c^	237	40.4
Glasses of water per day (number)^b^	586	4 (2, 6)	Soap availability change since COVID-19 pandemic^d^		
Drinking ≥8 glasses of water/day (%)	105	17.9	No, it is the same (%)	399	68.1
Water availability change since COVID-19 pandemic^d^			Yes, it got worse (%)	141	24.1
No, it is the same (%)	451	77	Yes, it improved (%)	46	7.8
Yes, it got worse (%)	108	18.4	Alcohol -based hand rub availability		
Yes, it improved (%)	27	4.6	Always (%)	157	26.8
Spent on water sources per month (USD)^b^	446	5 (2.5, 10)	Usually (%)	136	23.2
Main beverage when thirsty			Sometimes (%)	128	21.8
Regular water (%)	285	48.6	Rarely (%)	91	15.5
Soda (%)	207	35.3	Never (%)	74	12.6
Sweetened beverages (%)	46	7.8	Handwashing after a bowel movement		
Other beverages (%)	27	4.6	Always (%)	240	41
Alcohol beverages (%)	21	3.6	Usually (%)	158	27
Thirsty without water			Sometimes (%)	111	18.9
Multiple times per day (%)	71	12.1	Rarely (%)	60	10.2
Daily (%)	46	7.8	Never (%)	17	2.9
Multiple times per week (%)	149	25.4	Handwashing before eating		
Once (%)	104	17.7	Always (%)	193	32.9
Never (%)	216	36.9	Usually (%)	167	28.5
Sanitation	148	25.3	Sometimes (%)	134	22.9
Basic sanitation + availability (%)^c^	148	25.3	Rarely (%)	71	12.1
Basic sanitation (%)^a^*	167	28.5	Never (%)	21	3.6
Improved toilet facilities access (%)	456	77.8	Handwashing before preparing food		
Non-shared toilet facilities (%)	378	64.5	Always (%)	212	36.2
Toilet facility availability (%)^c^	351	59.9	Usually (%)	148	25.3
Expending ≤15 min to sanitation facility (%)	462	79.5	Sometimes (%)	117	20
Time to saniation facility (min)^b^	581	2 (0, 5)	Rarely (%)	64	10.9
Open defecation (%)	225	38.4	Never (%)	42	7.2
Times of open defecation per week^b^	225	5 (2, 7)	Bathing		
Toilet functionality (%)^c^	341	62.1	Bathing accessibility (%)*	267	45.6
Toilet privacy (%)^c^	286	50.4	Improved water source for bathing (%)	553	94.4
Experiencing violence/harassment using the toilet (%)	79	13.5	More than four bathes per week (%)	269	45.9
Spent on sanitation per month (USD)^b^	329	4 (1.5, 10)	Bathes per week (number)^b^	586	4 (2, 7)
Spent on WASH per month (USD)^b^	279	22.5 (10.5, 50)	Improved water source for cleaning wounds/abscesses (%)*	539	92
			Improved water source for preparing drugs for injection (%)*	551	94
			Spent on hygiene per month (USD)^b^	466	10 (5, 20)

*WASH* water, sanitation, and hygiene

^a^ Joint Monitoring Program definition

^b^ Median (range)

^c^ “Always”/24-hrs

^d^ March 2020

**Table 3:** Water sources in the last 6 months among PWID in 586 Tijuana-San Diego metropolitan area in 2020–2021

Water use and sources	n	%	Water use and sources	n	%
**Drinking**			**Bathing**		
Main water source			Main water source		
Improved water sources	542	92.5	Improved water sources	553	94.4
Packaged water	284	48.5	Private tap water	409	69.8
Private tap water	184	31.4	Public tap water	120	20.5
Public tap water	71	12.1	Other improved water sources	13	2.2
Other improved water sources	3	0.5	Packaged water	11	1.9
Unimproved water sources	2	0.3	Unimproved water sources	5	0.9
Surface water	8	1.4	Surface water	16	2.7
No water sources	34	5.8	No water sources	12	2.0
Use at least once			**Cleaning wounds and abscesses**		
Unimproved water sources	11	1.9	Main water source		
Surface water	19	3.2	Improved water sources	533	92.1
**Handwashing**			Private tap water	357	61.7
Main water source			Packaged water	100	17.3
Improved water sources	544	92.8	Public tap water	74	12.8
Private tap water	360	61.4	Other improved water sources	2	0.3
Public tap water	152	25.9	Unimproved water sources	4	0.7
Packaged water	19	3.2	Surface water	14	2.4
Handwashing station	10	1.7	No water sources	28	4.8
Other improved water sources	3	0.5	**Preparing drugs for injection**		
Unimproved water sources	22	3.8	Main water source		
Surface water	13	2.2	Improved water sources	556	94.9
No water sources	7	1.2	Private tap water	299	51
Use at least once			Packaged water	175	29.9
Unimproved water sources	30	5.1	Public tap water	58	9.9
Surface water	19	3.2	Sterile water	22	3.8
			Other improved water sources	2	0.3
			Unimproved water sources	12	2
			Surface water	13	2.2
			No water sources	5	0.9

*PWID* people who inject drugs

Only 28% of participants (32% in San Diego and 23% in Tijuana) reported basic sanitation access, and even fewer (25%) had basic sanitation access ‘always available’ (24-hours a day) (Table 2). Open defecation was reported by 38% of participants, with a median of five times (IQR: 2, 7) per week. Participants spent a median of 2 minutes (range from 0 min to 90 min) traveling to a sanitation facility and 20% spent more than 10 min. Toilet functionality and privacy was reported by 62 and 50% of the participants respectively. Experiencing violence or harassment using the toilet was reported by 13% of the participants (16% of women and 12% of men). Participants spent a median of $4 USD (IQR: $1.5, $50) on sanitation services per month.

Thirty-eight percent of the participants reported basic hand hygiene access, and 29% reported basic hand hygiene access with soap and improved water sources ‘always available’ (24-hours a day) (Table 2). For 68% of participants, soap availability did not change after the COVID-19 pandemic started, but it got worse for 24% of the sample population. More than half of the participants (61%) always or usually had alcohol-based rub available for hand hygiene practices. The main water source for handwashing was tap water (87%). Eight percent used surface water or unimproved water sources for handwashing at least once in the last 6 months. Always handwashing after bowel movements was reported by 41% of participants, before eating by 33%, and before preparing food by 36% of participants.

In terms of body hygiene practices, 46% of the participants reported bathing access (i.e., > 4 baths/week with improved water sources). Participants bathed a median of four times (IQR: 2, 7) per week and 94% used improved water sources (94%). Access to improved water sources for cleaning wounds or abscesses, and for preparing drugs for injection was greater than 90% (92 and 94% respectively). Tap water was the most common water source for bathing, cleaning wounds and abscesses, and for preparing drugs for injections (90, 79, and 81% respectively). Five percent reported no water sources for cleaning wounds and abscesses, and 4% used surface water or unimproved water sources for preparing drugs for injection. Participants spent a median of $10 USD (IQR: $5, $20), ranging from $0.50 to $250 USD on hygiene and $22.5 (IQR: $10.5, $50) USD, ranging from $2.3 to $405 USD on all WASH services per month.

### WASH access by City of residence and housing status

**Fig. 2:**
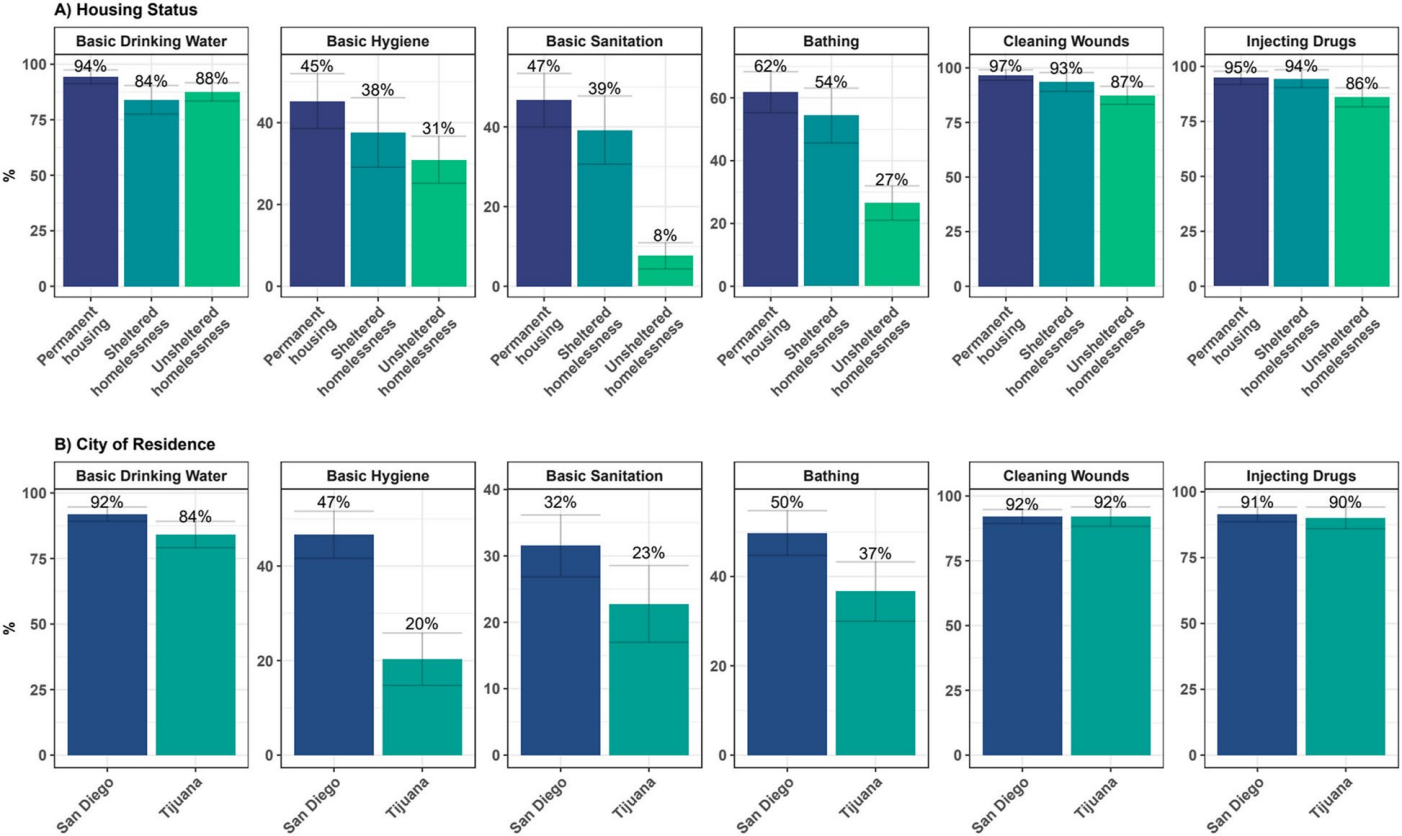
Access to WASH among 586 PWID in the Tijuana-San Diego metropolitan area by housing status and city of residence in 2020–2021. WASH – water, sanitation, and hygiene, PWID - people who inject drugs

San Diego (SD) residents had higher access to WASH services in comparison to Tijuana (TJ) residents (Fig. 2), especially in accessing basic hygiene (SD 55.8% vs TJ 30.0%) and sanitation (SD 43.1% vs TJ 20.0%). We observed a social gradient in WASH access by housing status across all key WASH access variables, except for access to basic drinking water. Participants living in permanent housing had higher access to key WASH services in comparison to people experiencing sheltered or unsheltered homelessness. These differences were notable between individuals living in permanent housing (PH) and those who experienced unsheltered homelessness (UH) in shower/bathing access (PH 79.2%, vs UH 30.9%), basic sanitation (PH 49.4% vs UH 12.7%), and basic hygiene (PH 57.1% vs UH 32.7%).

**Fig. 3:**
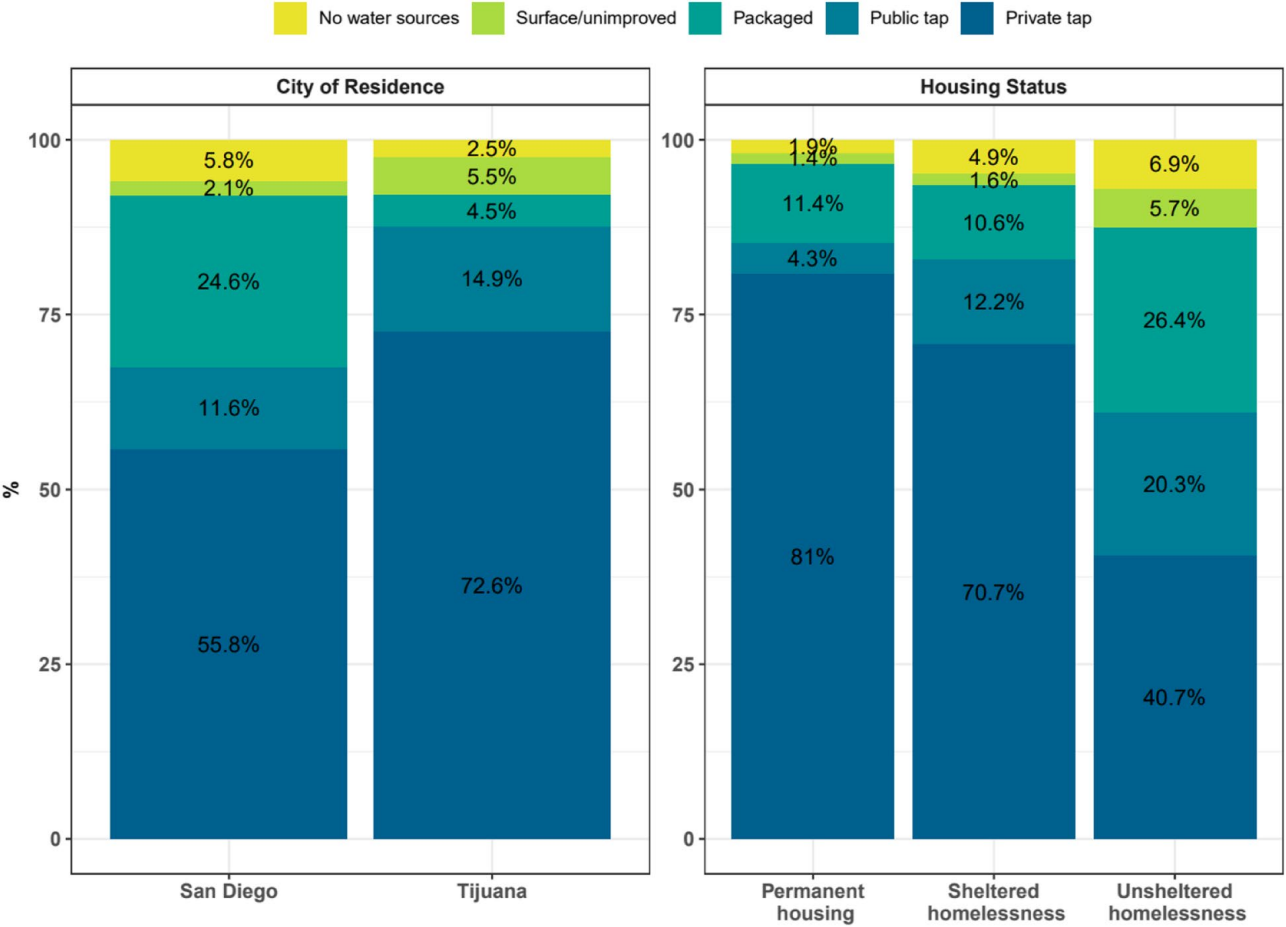
Main water sources for cleaning wounds and abscesses among 586 PWID in Tijuana-San Diego metropolitan area by housing status and city of residence in 2020–2021. PWID - people who inject drugs

About PWID-specific water need, public and private tap water were the most common water sources for cleaning wounds/abscesses in both cities (SD 81.2%, TJ 88.4%) (Fig. 3). About the same proportion of participants from both cities reported no water sources for cleaning wounds/abscesses (SD 3.0%, TJ 3.3%). Surface and unimproved water sources for this use were reported only by participants residing in Tijuana (5%). San Diego residents used more packaged water (15.8%) to clean wounds/abscesses than those living in Tijuana (3.3%).

Participants living in permanent housing used almost exclusively private tap water to clean wounds/abscesses (93.5%). Among individuals experiencing sheltered (SH) and unsheltered homelessness, private (SH 70.0%, UH 55.6%) and public taps (SH 10.0%, UH 14.8%) were the most common water sources for this use. Individuals experiencing unsheltered homelessness used more packaged water (20.4%) to clean wounds/abscesses than who were experiencing sheltered homelessness (13.3%) and those living in permanent housing (3.9%). More than 3% of individuals experiencing homelessness used surface or unimproved water sources for cleaning wounds/abscesses (SH 3.3%, UH 3.7%). Also, more than 3 % of sheltered (3.3%) and unsheltered (5.6%) individuals experiencing homelessness reported no water sources for these uses.

### Social and geographic factors associated with WASH insecurity

In multivariable analysis, participants who resided in Tijuana had 1.68 (aRR 95%CI: 1.02, 2.76) times more basic drinking water insecurity, 1.45 (aRR 95%CI: 1.28, 1.64) times more basic hygiene insecurity, and 1.21 (aRR 95%CI: 1.06, 1.39) times more bathing insecurity in comparison to those who resided in San Diego (Table 4). No significant differences were found in insecurity accessing sanitation and improved water sources for cleaning wounds or for preparing drugs by city of residence.

**Table 4:** Social and geographic factors associated with WASH insecurity variables among 586 PWID in Tijuana-San Diego metropolitan area in 2020–2021

Variable	Level	WASH insecurity indicator (%)	RR	95% CI	aRR	95% CI
Basic drinking water insecurity						
City of residence	San Diego	8.1				
	Tijuana	15.8	1.96	1.23, 3.12*	1.68	1.02, 2.76*
Housing status	Permanent housing	5.7				
	Sheltered homelessness	16	2.83	1.43, 5.58*	2.4	1.18, 4.90*
	Unsheltered homelessness	12.4	2.2	1.16, 4.17*	2.03	1.07, 3.86*
Basic sanitation insecurity						
City of residence	San Diego	68.5				
	Tijuana	77.2	1.13	1.02, 1.25*	1.07	0.97, 1.18
Housing status	Permanent housing	53.3				
	Sheltered homelessness	60.8	1.14	0.94, 1.38	1.13	0.93, 1.37
	Unsheltered homelessness	92.4	1.73	1.52, 1.98*	1.68	1.48, 1.92*
Basic hand hygiene insecurity						
City of residence	San Diego	53.4				
	Tijuana	79.7	1.49	1.33, 1.68*	1.45	1.28, 1.64*
Housing status	Permanent housing	54.7				
	Sheltered homelessness	62.4	1.14	0.95, 1.37	0.98	0.81, 1.18
	Unsheltered homelessness	69.1	1.26	1.09, 1.46*	1.15	0.99, 1.34
Bathing insecurity						
City of residence	San Diego	50.3				
	Tijuana	62.4	1.24	0.07, 1.44	1.21	1.06, 1.39*
Housing status	Permanent housing	37.7				
	Sheltered homelessness	45.6	1.21	0.93, 1.56	1.21	0.94, 1.57
	Unsheltered homelessness	73.1	1.94	1.60, 2.34*	1.84	1.52, 2.22*
Improved water for cleaning wounds/abscesses insecurity						
City of residence	San Diego	8.9				
	Tijuana	8.4	0.95	0.54, 1.65	0.85	0.49, 1.48
Housing status	Permanent housing	4.2				
	Sheltered homelessness	7.3	1.71	0.70, 4.19	1.93	0.78, 4.76
	Unsheltered homelessness	13.3	3.13	1.54, 6.40*	3.12	1.55, 6.29*
Improved water for preparing drugs insecurity						
City of residence	San Diego	8.6				
	Tijuana	9.9	1.15	0.68, 1.95	1.11	0.65, 1.88
Housing status	Permanent housing	5.2				
	Sheltered homelessness	5.6	1.08	0.43, 2.71	1.13	0.44, 2.86
	Unsheltered homelessness	14.1	2.71	1.41, 5.20*	2.58	1.36, 4.89*

Adjusted for gender and reported sex work in the past 6 months

* Statistically significant

Participants experiencing sheltered homelessness had 2.40 (aRR: 95%CI: 1.18, 4.90) times more insecurity for basic drinking water insecurity than those living in permanent housing. Participants experiencing unsheltered homelessness had 3.12 (aRR 95%CI: 1.55, 6.29) times more insecurity accessing improved water sources for cleaning wounds and abscesses and 2.58 (aRR: 95%CI: 1.36, 4.89) for preparing drugs for injection, 2.03 (aRR 95%CI: 1.07, 3.86) times more basic drinking water insecurity, 1.84 (aRR 95%CI: 1.52, 2.22) times more bathing insecurity, and 1.68 (aRR 95%CI: 1.48, 1.92) times more basic sanitation insecurity. No significant differences were found in hand hygiene insecurity by housing status.

## Discussion

This study provides the first comprehensive estimate of WASH access among PWID in the Tijuana–San Diego metropolitan area. Homelessness (sheltered or unsheltered) was common and was identified as the most significant intersectional vulnerability affecting access to WASH among PWID. Despite Tijuana and San Diego having profound socioeconomic differences – e.g., middle-income vs high-income country – the binational population of PWID faces similar challenges accessing WASH services on both sides of the border. Public WASH services are needed in both cities to reduce disparities in WASH access for PWID and to improve their health and wellbeing. Moreover, we developed variables quantifying access to ‘always available’ (24-hour) basic WASH services, which highlight accessibility gaps that are often ‘hidden’ in official data that do not consider the frequency of access.

### Drinking water

In our study, PWID access to basic drinking water was lower than the national averages in both countries (Mexico and the US report > 99% access). This is suboptimal as the international target is universal basic access [6]. Likewise, low quantity of water intake per day [29], feeling thirsty without available drinking water sources, and drinking soda and sweetened beverages instead of water increase PWID risk of metabolic diseases. Drinking water insecurity (i.e., lack of access) and suboptimal water intake can lead to acute or chronic dehydration (i.e., 1–2% loss of body water), impaired cognitive function (e.g., short term attention and memory loss), and urolithiasis and other kidney diseases in the long term [31]. Unsheltered homelessness status and residence in Tijuana were associated with basic drinking water insecurity, which is likely related to infrastructure inaccessibility (e.g., lack of public drinking fountains). A recent mixed methods study among PWID and who were experiencing unsheltered homelessness residing in Tijuana found that 40% reported dehydration in the week prior to the survey, attributing it to their limited access to drinking water sources [16]. Similarly, a study among people experiencing homelessness – many who use substances – in San Diego found that increased distance from sources of safe drinking water may heighten risk of dehydration [23]. Our study complements previous work, highlighting the severe water insecurity and dehydration risk among this population of PWID.

### Sanitation

According to international monitoring data, basic sanitation access in urban areas has been described as 100% for the US and 94% for Mexico, and both countries report that fewer than 1% of the population practice open defecation [6]. Despite residing in metropolitan areas that are each high-income relative to the country in which they are located, participants’ access to basic sanitation was very low (< 30%) and open defecation was a common practice (~ 40%). The Project for Sanitation Justice in San Diego reported in 2022 that 49% of census tracts don’t have public sanitation facilities, which are often closed or locked and almost none are open 24 hours [32]. Although no similar information is available for Tijuana, access is expected to be similar or more limited. We identified that participants who did have access to toilets reported that the toilets were often non-functional, without privacy (i.e., toilet with door that locks), and frequently experienced violence using them. Further, basic sanitation insecurity was more challenging among individuals experiencing unsheltered homelessness in both cities, increasing their risk of infectious diseases. Particularly, open defecation poses risks not only to PWID, but is a major public health risk in both cities due to fecal contamination in soil and surface water. For instance, unhoused individuals with insufficient access to sanitation services may be unable to avoid contributing to human fecal contamination of the environment where they live and the Tijuana River and San Diego River Watersheds pollution [22, 33]. River water contamination can further affect environmental health during storm events and when untreated water reaches ocean and estuary environments [34, 35]. Increases in 24-hour public restroom access, especially in neighborhoods with people experiencing homelessness could therefore have a large community health impact by reducing open defecation [36]. In the Tijuana River, fecal contamination is also contributed to by untreated sewage and lack of wastewater treatment capacity. Fecal contamination was high immediately upstream of the location where the community who lives inside the river canal resides. In the case of the Tijuana River, the lack of capacity in the wastewater treatment system contributes to untreated wastewater discharges, especially during rain events, which has been described as the most important source of human contamination [22, 37, 38]. Similarly, in the San Diego River, researchers found chemical markers indicating that high levels of fecal contamination during storm events are primarily from leaky sewers and not open defecation [39].

### Hygiene

Official data in Mexico reports that 91% of individuals have access to basic hand hygiene, and although there are no official data for the US, nearly universal access is expected [6]. Yet, PWID participating in this study were far from achieving this national access to basic hand hygiene. Basic hygiene access insecurity could be limiting hygiene practices at key moments (i.e., after a bowel movement, before eating, and before preparing food). Additionally, although the use of unimproved and surface water sources was not common, they did represent the main water source for hand hygiene for some participants. A study in Zimbabwe reported that contaminated water for handwashing was associated with hand contamination with *E. coli* [40]*.*

In this study we described bathing access based on the number of baths per week and the water sources used. However, the location or facilities this population used for bathing remain unclear from this study. Different studies among PWID and who experience unsheltered homelessness residing in Tijuana and San Diego have reported business establishments (~ 30%) such as private stores or gas stations [16, 23] were the most common places to bathe, which have sinks and do not have showers or baths. Considering that our study population could share this practice, we are unsure that bathing access was from a proper facility – e.g., different than a sink in a public restroom, and this issue should be further studied.

### PWID-specific water needs

Although most PWID participants reported using improved water sources for drug injection preparation and for cleaning wounds and abscesses, the use of surface water and other unimproved water sources remained present (5–8%). This is an important finding because the lack of safely managed water sources for these behavior practices may exacerbate health risks, including the re-occurrence of abscesses among the population. In different contexts, abscesses – and other skin and soft tissue infections (SSTI) – are common (~ 50%) among PWID [13, 16, 23], which highlights the need to conduct more research on water sources associated with SSTI among PWID. Particularly among PWID and who experienced unsheltered homelessness residing in the Tijuana, SSTI were significantly associated with use or contact with contaminated surface water [16]. A study of people experiencing unsheltered homelessness who used drugs in rural areas of Central Appalachian Kentucky supported the notion that harm reduction services providing clean water for preparing drugs, cleaning skin, and handwashing facilities prior to injection would help prevent bacterial infections and abscesses among PWID and who experienced unsheltered homelessness [12]. Harm reduction programs can provide PWID with access to WASH services and education about hygiene practices around drug preparation [17], decreasing PWID’s risk of SSTI and water-related infectious diseases.

### Geographic inequalities

Despite Tijuana being one of the most developed cities in Mexico [41], it does not have the same WASH infrastructure and availability of free public services for residents that San Diego offers. This includes access to public drinking fountains, sanitation and hygiene facilities at public spaces, and mobile hygiene services.

In San Diego, after the Hepatitis A outbreak in 2017 and during the COVID-19 outbreak in 2020, the local government set up temporary handwashing stations and portable toilets for individuals experiencing unsheltered homelessness [23, 42]. However, study staff in San Diego observed that many toilets in public spaces (e.g., parks) were locked or unavailable outside of daylight hours. In contrast, in Tijuana during the COVID-19 outbreak in 2020, local businesses and nonprofit organizations provided handwashing stations; yet few public facilities were established, which may explain geographic differences on basic hand hygiene insecurity.

Further, differences between both cities in infrastructure and availability of programs for individuals experiencing homelessness may explain bathing inequalities. About half of the population experiencing homelessness residing in the San Diego River used restroom sinks at businesses, portable toilets or public restrooms to bathe, and the remainder used service providers, shelters, homelessness service facilities, or saved up money to stay in a hotel room once per month to shower [23]. On the other hand, among the PWID and who experienced unsheltered homelessness residing in the Tijuana River, few bathing facilities were available, and there was a higher reported levels of contact with unimproved water sources, such as surface water – including irrigation water (clean tap water in Mexico), or purchased packaged water [16]. Furthermore, relative to their income (participants in Tijuana had lower income than in San Diego), many PWID in this region spent a large quantity of money on WASH services every month, especially on hygiene supplies. Although there are differences between Tijuana and San Diego that require tailoring an approach to each context, we found few differences in term of WASH access suggesting that policy responses should be similar.

### Social inequalities

WASH access is integrally connected to shelter/housing. PWID and who experienced unsheltered homelessness had significantly higher experiences of WASH insecurity than those who lived in permanent housing. Lack of access to private tap water sources – linked with housing access – among participants experiencing unsheltered homelessness may be related to the higher use of packaged water. In many cases water bottles and sterile water were provided by harm reduction services and were the only water source for preparing drugs for injection and cleaning wounds for the sampled population. Intersectional vulnerabilities, like experiencing unsheltered homelessness, exacerbate WASH insecurity in distinct fashions for both men and women that prevent them from participating in daily activities [8, 43].

Sanitation and bathing facilities are services that require infrastructure that is less accessible outside a formal housing setting. In two studies of communities experiencing homelessness in Los Angeles, CA and Belo Horizonte, Brazil, during the night, public toilets and bathing facilities are usually closed and open defecation becomes a coping survival strategy [8, 44]. Many individuals in the Brazilian study reported urinating and defecating in the open near where they sleep [44], avoiding violence, but increasing environmental risk for themselves and others around them. Individuals experiencing homelessness are at higher risk of WASH insecurity, including people experiencing sheltered homelessness, which can be living in hotel rooms, garages, or *cuarterias* (building divided into small informal living spaces) where bathrooms are not always available. Furthermore, WASH insecurity perpetuates a cycle of poverty among populations experiencing unsheltered homelessness [8], and criminalization of homelessness in both cities also contribute to experiences of WASH insecurity [16, 23]. Particularly hygiene insecurity (bathing and handwashing) can exacerbate social exclusion, discrimination, police victimization, and loss of dignity and self-esteem.

Our findings lead us to propose two possible avenues to achieve WASH security among populations of PWID and who experienced unsheltered homelessness in our study population: 1) a *housing first* (HF) model where WASH is available within the household setting, and 2) mobile/public WASH facilities to meet all other WASH needs. HF principles include immediate access to affordable housing, and independent individualized and flexible support [45]. Mobile WASH facilities can help by bringing WASH services to PWID who are experiencing homelessness. In San Diego there are public and nonprofit mobile hygiene services available that provide shower services and hygiene supplies to populations experiencing homelessness, while in Tijuana these services are limited to a small number of nonprofit organizations. Furthermore, to access sanitation services, in Tijuana (as in many other locations in Mexico) there is a cost incurred for use of public bathrooms (~$0.25–$0.5 USD) and in San Diego, the San Diego City Council is considering charging for public restrooms as well, limiting the accessibility for anyone experiencing homelessness or who have scarce financial resources [46].

### Contributions to JMP definitions

Current JMP definitions of basic WASH access have two implications for the population of PWID studied. First, the unit of measure is the ‘household’, which excludes individuals experiencing homelessness. Second, this definition does not include data on temporal availability of these facilities, therefore we do not know whether sanitation facilities are ‘always available.’ We report access using the JMP definitions and additionally described an extended version of them, including 24-hour availability of WASH services. Particularly, for basic hand hygiene access, we also incorporated availability of soap and the use of improved water sources, no guarantee among marginalized populations. The proposed extended definitions highlight the accessibility gaps hidden in basic WASH access definitions, which is particularly important among communities experiencing homelessness that use public facilities to solve daily needs. There are other scales to measure water insecurity [47], yet we defined WASH insecurity indicators in a simple way based on JMP definition for basic WASH access, highlighting the need to include sanitation and hygiene insecurity, which are usually left out.

In addition to the JMP water quality standards for drinking water, we extended the JMP water sources definitions for handwashing, bathing, and PWID-specific water needs. Moreover, WASH literature has typically focused on hand hygiene but not on bathing. More data should be collected on bathing, including frequency, water sources, facilities, soap/shampoo availability, and health outcomes.

### Limitations

Due to a restricted sample size, we could not analyze intersectional interactions of WASH insecurity between more than one social and geographic stratifier at time. There was also potential selection bias based on the significance testing for those included vs excluded, though excluded participants were < 0.05% of the parent study sample, and also because participants residing in San Diego who engaged in cross-border drug use were oversampled since the goal of the parent study was to study risks of infectious diseases and overdose among PWID in relation to cross-border mobility. This study was based on a self-reported questionnaire about WASH access, which is prone to recall and response biases if participants could not accurately remember details or if they tended to respond with what they believed was a socially desirable answer. Social desirability bias may have led some behaviors, like open defecation, to be under-estimated. The WASH questionnaire also included questions that can feel private, such as handwashing and open defecation practices. We did not include questions about the facilities used for bathing. Additionally, access to laundry services, oral hygiene, and water sources for rinsing syringes and handwashing before injection practice are important variables that could be collected in future research but were not explored in this study. Future efforts to implement mobile WASH services or other interventions should involve PWID to ensure that the services meet their needs.

## Conclusions

WASH access among PWID in the Tijuana-San Diego metropolitan area was low by international standards and much lower than national averages in both countries. Even in one of the highest income countries in the world, marginalized populations of PWID had extremely poor access to basic WASH services. Homelessness was frequent and significantly associated with WASH insecurity among this population. Concentrated efforts are needed to ensure WASH access for PWID, especially among people who experience unsheltered homelessness, in this urban border region. Continuously available (24-hour) basic WASH services are important among PWID experiencing homelessness and should be evaluated in further studies. Harm reduction programs are pivotal sites for unsheltered homeless PWID, and their expansion could help address unmet WASH needs in the Mexico-US border region. Additionally, global WASH assessment surveys should be inclusive of populations experiencing homelessness to address availability and need of basic WASH services.

### Supplementary Information

** Additional file 1: Supplementary Table 1.** Characteristic of excluded and included *La Frontera* participants, 2020–2021.

## Data Availability

The data used in this paper are components of the doctoral thesis for the first author which has yet to be completed. Requests for access can be made to Dr. S. Strathdee.
